# Case report: Diagnosis and surgical treatment of delayed traumatic diaphragmatic hernia with hepatothorax and enterothorax in a small dog

**DOI:** 10.3389/fvets.2024.1357626

**Published:** 2024-06-14

**Authors:** Bing Shao, Yiding Liu, Tiange Tai, Zhaoyang Liu, Tianyu Han, Yu Yang, Shanshan Fei, Shu Wang, Haibin Wang, Tiezhu Chen, Guangliang Shi

**Affiliations:** ^1^Heilongjiang Northeast Agricultural University Animal Hospital Company Limited, College of Veterinary Medicine, Northeast Agricultural University, Harbin, China; ^2^Sichuan Academy of Chinese Medicine Sciences, Chengdu, China; ^3^Sichuan Provincial Key Laboratory of Quality and Innovation Research of Chinese Materia Medica, Chengdu, China

**Keywords:** dog, delayed diaphragmatic hernia, X-ray, ultrasound examination, case report

## Abstract

An 8-year-old castrated male teddy bear dog presented to our clinic with a persistent cough. The sick dog suffered from vehicular trauma 6 months prior to the visit and had imaging and exploratory laparotomy. Imaging and exploratory laparotomy at the time showed no significant damage. We performed contrast radiography (barium gavage) on the sick dog. Based on the results of a complete contrast radiography (barium gavage), tubular shadows in the thoracic cavity were identified as the small intestine and cecum, and delayed traumatic diaphragmatic hernia with hepatothorax and enterothorax was confirmed with radiographs. Accordingly, the sick dog underwent general anesthesia, manual ventilation and diaphragmatic herniorrhaphy by standard ventral midline abdominal approach. Postoperatively, the dog was given analgesia and antibacterial treatment, and the liver biochemical indexes were monitored to prevent endotoxin. Postoperative radiographs revealed clear contours of thoracic and abdominal organs. The dog moved, ate, and urinated normally within 10 days of the surgery. This case provides a reference for a complete barium meal imaging procedure that clearly shows the position of the organs in the thoracoabdominal cavity after the occurrence of a delayed traumatic diaphragmatic hernia. This paper provides a practical reference for the diagnosis of delayed traumatic diaphragmatic hernia with hepatothorax and enterothorax.

## 1 Introduction

Diaphragmatic hernias, common in cats and dogs, can be congenital or traumatic. Congenital diaphragmatic hernia is characterized by a musculoskeletal defect, manifested as cracks in the diaphragm tendon or muscle portion, allowing abdominal organs to enter the chest cavity (1). This hernia is difficult to diagnose because small animals with congenital diaphragmatic hernia have almost no clinical symptoms, most often being an incidental finding during necropsy or diagnostic imaging (2). Traumatic diaphragmatic hernias occur following blunt force trauma to the abdomen causing increased abdominal pressure. When this force is combined with an open glottis, the air-filled lungs deflate, causing an increased pressure gradient across the diaphragm. This sudden pressure change across the diaphragm causes it to rupture (3). Vehicular trauma are the most common cause of traumatic diaphragmatic hernia, which presents through varied clinical signs, with dyspnea being the most common (4).

Delayed diaphragmatic hernia is a long-term complication of an undetected diaphragmatic injury that occurs weeks or even years after an injury and is not accompanied by diaphragmatic hernia manifestations in the acute phase (5, 6). Delayed diaphragmatic hernia is less common and can present with varied symptoms, including cough, dyspnea, abdominal pain, diarrhea, vomiting, and in severe cases, respiratory failure or cardiopulmonary failure (7). Hepatothorax is the displacement of the liver into the thorax due to a traumatic rupture of the right diaphragm. The main clinical presentation is usually dyspnea and abdominal pain, but may also include cyanosis, arrhythmia, and hypotension. Surgical repair is the standard treatment for diaphragmatic hernia (8). The current standard treatment for diaphragmatic hernia repair in small animals is laparotomy and thoracotomy. The approach depended on the hemodynamic stability of the patients, and the preference and skills of the surgeon (9). In addition, when delayed diaphragmatic hernia occurs, if adhesions between visceras and chest is suspected, thoracotomy or combined thoracic-abdominal approach is preferred.

This report describes the clinical examination, imaging results, and surgical procedure for delayed traumatic diaphragmatic hernia with hepatothorax and enterothorax in an 8-year-old dog. The case underwent a comprehensive barium meal imaging examination, and for the first time the specific condition of the gastrointestinal tract in the thoracoabdominal cavity after the occurrence of delayed diaphragmatic hernia was carefully documented. The aim of this case report is to provide a reference for identifying and treating similar cases.

## 2 Case description

Teddy bear dog, a male (de-sexed), 8-year-old dog weighing 3.9 kg, was involved in a vehicular trauma 6 months prior to his presentation to our facility. The dog had undergone exploratory laparotomy at the time, and no apparent visceral damage was observed. Before coming to our hospital for treatment, the dog exhibited a severe cough for 1 month. The coughing was only temporarily relieved with azithromycin, but occasional coughing was still present.

The sick dog was in good mental condition, rectal temperature was 38.6°C, the heart rate was 104 beats/min, both forelimbs dilated outward, the chest was exposed, and the chest was sensitive to palpation and showed signs of pain. Blood routine examination showed no obvious abnormality. The platelet count was 541 10^9^/L, which was beyond the normal range (the reference ranges: 117–490 10^9^/L), indicative of chronic infection. Blood biochemical tests (Supplementary Figure S1) revealed that alkaline phosphatase (ALKP) was 317 U/L (the reference ranges: 20–150 U/L), which was more than twice the normal level, and alanine aminotransferase (ALT) was 1,012 U/L (the reference ranges: 10–118 U/L), which was nearly nine times the normal level, suggestive of liver damage.

**Figure 1:**
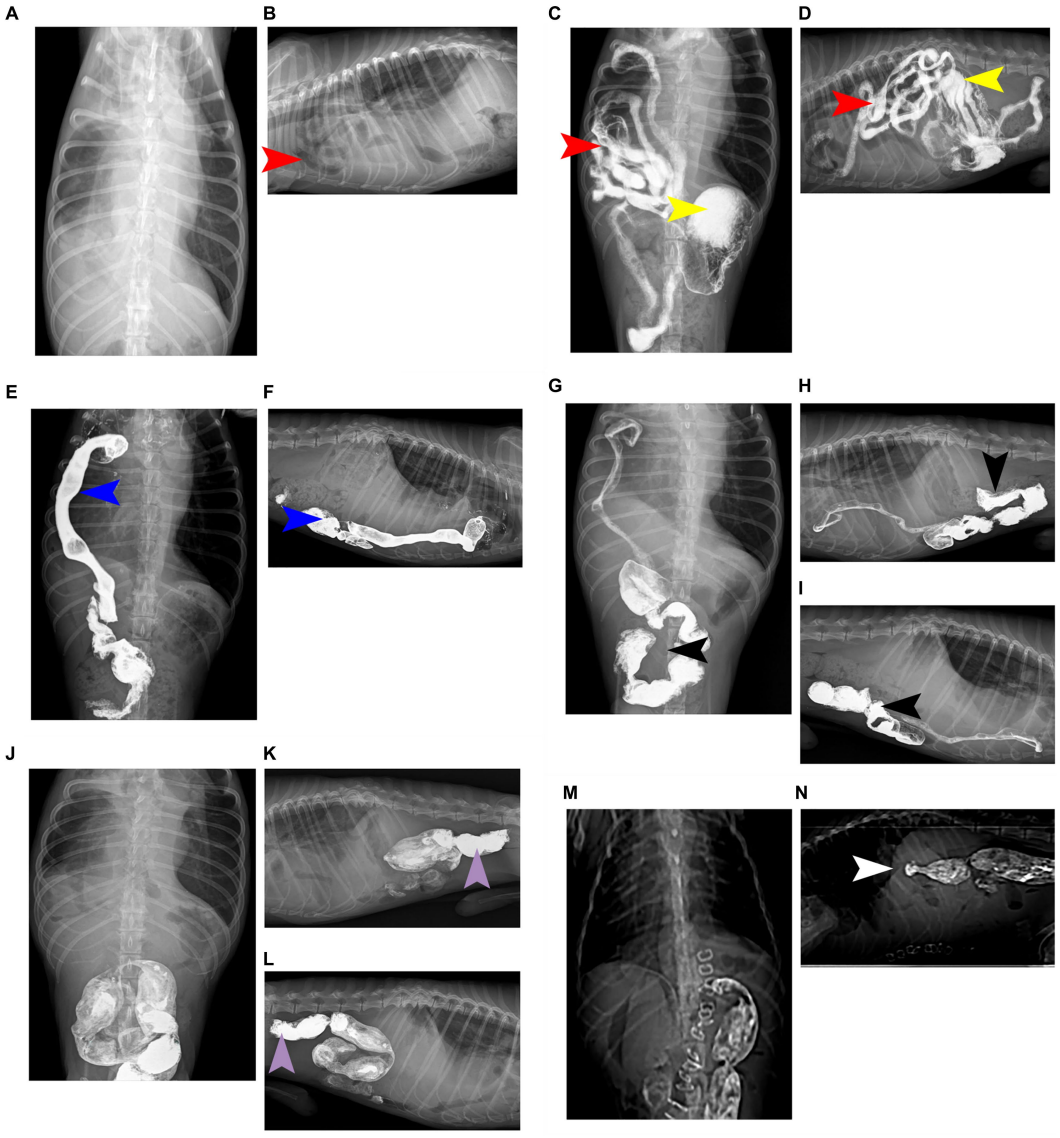
Complete barium meal contrast procedure. **(A)** Oral barium preprandial chest and abdominal dorsal radiographs showed abnormal shadows with uneven density and air-containing cavities in the chest. **(B)** Oral barium prepristine chest right position radiograph, showing a low-density tubular shadow in the chest cavity and a clear contour of the tube wall. **(C)** 30 minutes after oral barium meal, a chest and abdominal dorsum X-ray shows a large amount of barium meal deposition in the stomach, with some barium meal entering the small intestine, which is located in the right chest cavity. **(D)** 30 minutes after oral barium meal, the right chest X-ray shows the appearance of small intestine in the chest cavity, with barium meal deposition and high-density imaging. **(E)** After 2 hours and 30 minutes of oral barium meal, a chest abdominal and dorsal X-ray shows that the barium meal enters the cecum and is located in the right chest cavity, presenting high-density imaging. **(F)** On the right chest X-ray 2 hours and 30 minutes after oral barium meal, it can be seen that the barium meal enters the cecum and is located in the chest cavity, showing a high-density effect. **(G)** 4 hours and 30 minutes after oral barium meal, there is a small amount of barium meal located in the cecum, and most of the barium meal enters the colon and is located in the abdominal cavity, presenting high-density imaging. **(H)** On the right chest X-ray 4 hours and 30 minutes after oral barium meal, a large amount of barium meal can be seen entering the colon and located in the abdominal cavity. **(I)** On the left chest X-ray 4 hours and 30 minutes after oral barium meal, a large amount of barium meal can be seen entering the colon and located in the abdominal cavity. **(J)** 21 hours and 30 minutes after oral barium meal, there is no residual barium meal in the chest and intestines, and barium meal enters the colon and rectum, presenting high-density imaging. **(K)** 21 hours and 30 minutes after oral barium meal, on the right chest X-ray, it can be seen that there is no residual barium meal in the intestinal tract of the chest. The barium meal enters the colon and rectum, located in the abdominal cavity. **(L)** On the left chest X-ray 21 hours and 30 minutes after oral barium meal, it can be seen that there is no residual barium meal in the thoracic intestine. The barium meal enters the colon and rectum, located in the abdominal cavity. **(M)** Postoperative chest and abdominal dorsal radiograph, it can be seen that the chest cavity and abdominal organs have clear contours and obvious diaphragm. **(N)** Postoperative chest right radiograph, the chest cavity and abdominal organs are clearly contoured and the diaphragm line is obvious. The organ indicated by the red arrow is the small intestine; the organ indicated by the yellow arrow is the stomach; the organ indicated by the blue arrow is the cecum; the organ indicated by the black arrow is the colon; the organ indicated by the purple arrow is the rectum; and the part indicated by the white arrow is the diaphragm line.

Radiographs revealed a low-density tubular shadow in the thoracic cavity with a well-defined wall (Figures 1A,B). We chose the barium sulfate powder provided by the manufacturer, which was mixed with water to prepare a 60% w/v barium sulfate suspension (Barium Sulfate (Type II) for Suspension, Shandong Shengli Pharmaceutical Co., Ltd., China). The suspension was administered via an orogastric tube at a dose of 15 mL/kg (10). After 30 minutes, the dogs was examined by X-ray again, radiograph revealed that the tubular shadow in the chest cavity contains a large amount of barium sulfate suspension (Figures 1C,D). The sick dog was diagnosed with diaphragmatic hernia, and the small intestine entered the thoracic cavity through the hernia hole. Radiographs were taken 2 hours and 30 minutes after the oral gavages with barium, and barium deposits were seen in the cecum, which was located in the thoracic cavity, confirming the herniation of the cecum into the thoracic cavity (Figures 1E,F). At 4 hours and 30 minutes after the oral gavages with barium, radiographs of the chest of the sick dog in the dorsal, right and left positions was performed, and a small portion of the barium was observed in the cecum, but not the thoracic cavity. Furthermore, most of the barium went into the colon and rectum, which was located in the abdominal cavity (Figures 1G–I). Radiographs of the dorsal, right lateral and left lateral positions of the chest of the sick dog were taken 21 hours and 30 minutes after oral gavages with barium. The results showed that all the barium had moved to the colon and rectum, both of which were in the abdominal cavity (Figures 1J–L).

**Figure 2:**
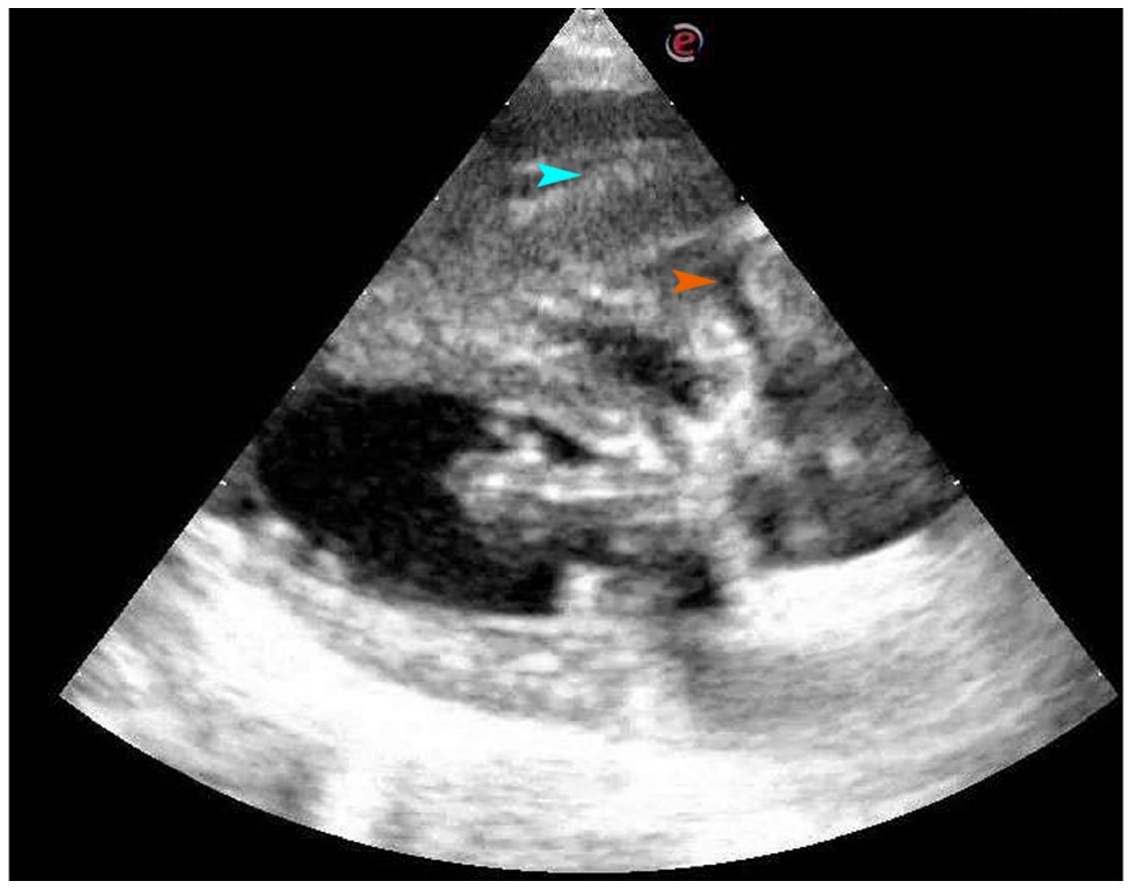
Four-chamber view of long axis of right sternum. On the ventral side of the heart, there is a soft tissue mass, which is the right liver lobe, and a small amount of liquid area can be seen between the myocardium and the soft tissue image. The organ indicated by the blue arrow is the liver, while the part indicated by the orange arrow is fluid accumulation.

An ultrasound examination of the ventral side of the heart revealed soft tissue in the right hepatic lobe with myocardial diastolic obstruction and a small area of fluid between the myocardium and the soft tissue image, which further increased the suspicion of diaphragmatic hernia (Figure 2).

Since no abnormality was found 6 months prior when the dog was involved in an accident, a delayed traumatic diaphragmatic hernia was initially considered. Based on the clinical signs of the sick dog, combined with blood routine examination, blood biochemical examination, ultrasonic examination, and contrast radiography was performed. The results showed small intestine and cecum in the thoracic cavity, and ultrasound showed liver and fluid area on the ventral side of the heart. A further comprehensive analysis confirmed the presence of delayed traumatic diaphragmatic hernia with hepatothorax and enterothorax, and the contents of the hernia were the small intestine, cecum and liver.

**Figure 3:**
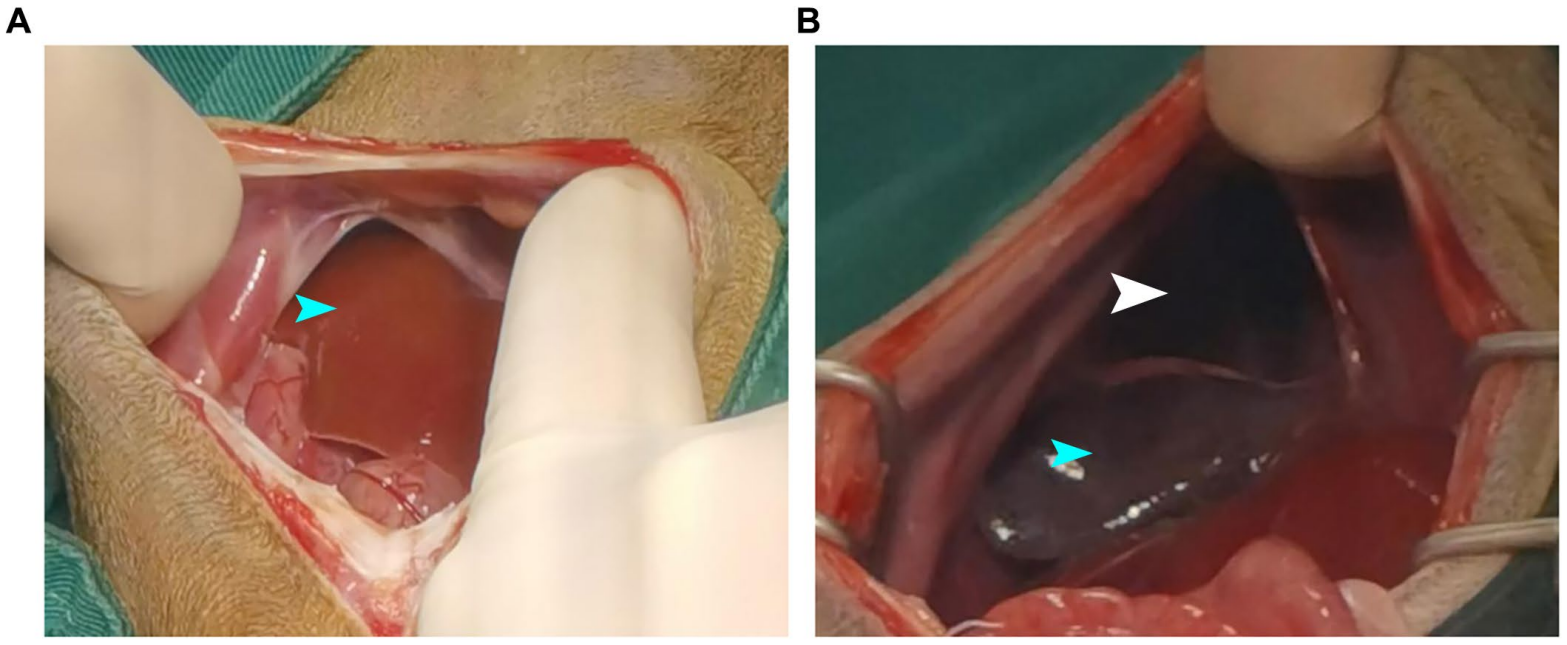
Showing intraoperative findings. **(A)** Liver and intestinal herniation into the chest cavity. **(B)** The liver lobes are purplish-red and hernia holes are visible. The organ indicated by the blue arrow is the liver, while the part indicated by the white arrow is the hernia.

Meloxicam (0.2 mg/kg SC, Metacam, Boehringer Ingelheim/Rhein Germany) and ceftiofur sodium (7 mg/kg IV, Woruite, YUANZHNG/China) were administered prior to surgery. Stabilization was initialized with oxygen supplementation by mask with a flow rate of 2 L/min. After induction with propofol (4 mg/kg IV, Propofol Injection, JIABO/China, 10 mg/mL), the patient was intubated with an endotracheal tube and maintained with isoflurane (Isoflurane, Ringpu/China) in oxygen. Lactated Ringer’s solution was administered (10 mL/kg/h, IV) throughout anesthesia. The dog was moved to the operating room, positioned in dorsal recumbency, and connected to an anesthetic machine. The sick dog was mechanically ventilated by use of a respirator operated. The respiratory rate was adjusted to maintain an ETCO_2_ of 35–45 mm Hg. Initial vaporizer setting was 1.5% isoflurane. Median expired isoflurane concentration was 1.2% during the surgery. Intermittent positive pressure ventilation was started. Minute ventilation was continuously monitored throughout anesthesia. A skin incision of approximately 8–10 cm was made along the midline of the abdomen posterior to the xiphoid prominence, and the subcutaneous tissue was separated to expose the abdominal cavity. Part of the liver and intestine could be seen, and an abnormal intestinal course was seen by running down the intestine. Further exploration revealed that part of the small intestine and liver lobe had entered the thoracic cavity, and a hernia hole were seen on the right diaphragm (Figure 3A). The abdominal wall retractor was used to prop up the abdominal cavity and expand the field of view. First, the intestine that had entered the thoracic cavity was slowly removed and retracted outside the body, followed by the move out of the liver. The liver lobe in the thoracic cavity was purplish-red, the hernia hole was visible at this stage (Figure 3B), and the diaphragm had a circular tear hole. A small amount of milky fluid was found in the thoracic cavity, which was absorbed with gauze. The thoracic cavity was flushed with warm saline, and the saline residue was aspirated with gauze. The thoracic and abdominal organs were examined for ischemia or other injuries. Each organ was intact before the hernia hole was closed and the ruptured diaphragm hole was repaired. Starting from the sternum, the hernia hole was closed with SIS (simple interrupted suture), and after the last stitch, a slit was left to insert an extension tube. The surgical assistant used a syringe to extract the residual liquid and gas in the chest cavity to restore the negative pressure in this cavity to prevent the occurrence of postoperative complications similar to pulmonary atelectasis and pulmonary atrophy. The intestine and liver were reset, and the abdominal cavity was closed. Postoperative chest plain radiograph results showed good diaphragm continuity, and no abdominal organs, such as the intestine and liver, were seen in the thoracic cavity (Figures 1M,N). Care was focused on protecting the liver, replenishing energy, protein, and electrolytes, maintaining acid–base balance in the body, preventing antibacterial infection, pain relief, and modulating inflammation. Postoperatively, the dog was treated with intravenous maintenance fluids (2 mL/kg/h; NaCl 0.9%, Braun). Postoperative analgesia was provided with a constant rate infusion of lidocaine (2 mg/kg/h, Lidor, Richter Pharma) IV. Meloxicam 0.1 mg/kg SC q24h (Metacam, Boehringer Ingelheim), and ceftiofur sodium (7 mg/kg IV q8h, Woruite, YUANZHNG/China) were administered during recovery.

After 4 days of continuous administration (include analgesia and antimicrobial therapy), the dog fed normally and had normal bowel movements and normal urination. There was a painful reaction during deep palpation in the sick dog. PLR test was positive. However, the dog was in good mental condition on day 10 after the operation and moved, ate, and urinated normally.

## 3 Discussion

**Figure 4:**
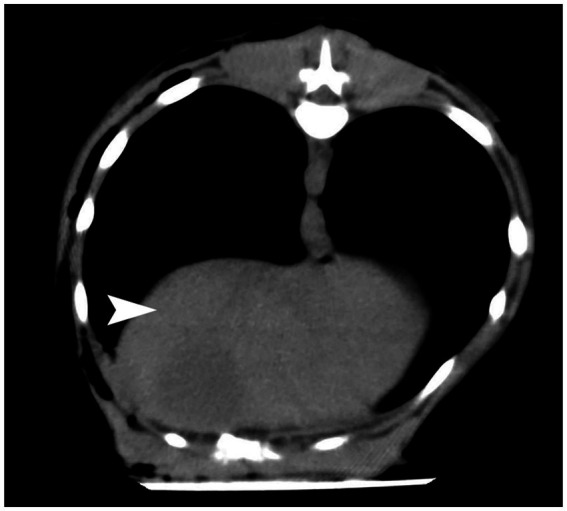
CT results after a vehicular trauma in October 2022.The diaphragm was seen to be in good continuity from the CT images with no obvious damage. The part indicated by the white arrow is the diaphragm.

The present case report presents a case of a diaphragmatic hernia in a dog after a vehicular trauma. Diaphragmatic hernia is most commonly seen in the pleural and refers to the protrusion of abdominal visceral organs into the thoracic cavity through a natural or traumatic diaphragmatic fissure. The clinical manifestations of delayed diaphragmatic hernia are not specific (11), but include (1) delayed rupture of the diaphragm, partial rupture of the diaphragm in the acute phase, or complete rupture of the diaphragm due to pressure difference in the thoracoabdominal cavity or local infection of the diaphragm; (2) chronic diaphragmatic hernias: the rupture in the diaphragm is small, and the abdominal organs herniate gradually and slowly; (3) missed diagnosis of diaphragmatic rupture arising from lack of consultation in the acute phase, lack of imaging examination, or missing out on no abnormalities in imaging findings (12). The last scenario is what was witnessed in this report, in which no obvious diaphragmatic rupture was ever seen in the chest CT examination after a vehicular trauma (Figure 4). It was not until 6 months later that the dog began to cough frequently, but there were no other clinical symptoms. It has been suggested that in traumatic diaphragmatic hernia, the site of diaphragmatic hernia is more common on the left side for the following reasons: firstly, the left side of the diaphragm is relatively weaker than the right side, and this is related to the good fusion of the right diaphragm during embryonic development; secondly, the right diaphragm is cushioned by the liver during external impact (13), which minimizes the risk of tear. Thus, the location of diaphragmatic rupture is related to both the location of the external impact and the position of the dog at the time of impact (14). The organs that herniate depend on the location of the diaphragm rupture, and the herniation varies with the organs involved. Right-sided diaphragm rupture often results in the herniation of the small intestine and pancreas, while left-sided diaphragm rupture often results in the herniation of the stomach, small intestine, and spleen (15). The clinical symptoms of traumatic diaphragmatic hernia are mainly related to the displacement of internal organs and manifest in three areas: the lung, heart, and gastrointestinal tract. Furthermore, they mainly involve, dyspnea, pain in the abdomen, and cyanosis of the conjunctiva, mouth, and tongue. In the case of delayed diaphragmatic hernia, the most important thing is to confirm the diagnosis as early as possible and perform the surgery in time, which will help in the postoperative recovery (16).

Diagnosis of diaphragmatic hernia is mainly performed by conventional X-ray, contrast radiography (barium gavage) and ultrasonography (17). Chest radiograph may show the disappearance of the diaphragmatic outline, fluid or air-filled organs in the thoracic cavity, as well as the disappearance of normal lung fields, and an abdominal radiograph may show the absence of abdominal organs (15). In the present case, X-ray and echocardiography were used to confirm the diagnosis. The sick dog underwent a 24-h contrast radiography (barium gavage) examination, which fully and clearly displayed the gastrointestinal condition of the sick dog. Before administration of barium sulfate suspension through orogastric tube, chest radiograph showed low-density tubular shadows in the chest cavity. In order to further diagnose and understand the patient’s condition in detail, a contrast radiography (barium gavage) examination was performed. The results showed that some small intestine and cecum entered the chest cavity, which was consistent with the X-ray examination of the above diaphragmatic hernia. The echocardiographic findings showed soft tissue images and a small amount of fluid on the ventral side of the heart, while laboratory findings showed elevated ALT, which is mainly secreted hepatocytes. Damage to hepatocytes or a change in the permeability of hepatocytes’ membranes due to liver disease causes an increase in the blood ALT level, and the level of ALT increase is proportional to the liver damage (18). Thus, a high ALT level is a specific indicator for hepatocyte damage. Based on the normal CT examination results of the dog after the vehicular trauma last year, the comprehensive diagnosis is delayed right diaphragmatic rupture in the dog. The normal physiological functions of the lungs and heart are affected by the compression of the liver and intestines, resulting in respiratory symptoms, including the inability of the heart to fully dilate, resulting in reduced blood supply, exercise intolerance, and decreased stamina. In the present study, the right lobe of liver entered the thoracic cavity through a hernia. The right lobe of liver had a right hepatic vein on it, affecting blood circulation. However, no other liver abnormality indices were explored. Combined with the purplish-red coloration of the right inner lobe of the liver, the elevated ALT was considered to be caused by blood circulation disorders, and the liver function had to be monitored at a later stage to prevent the production of endotoxins (19). Slight congestion due to blood circulation disorders was observed in the intestines. In the clinical field of small animals, barium sulfate has always been considered an important tool for diagnosing gastrointestinal diseases. Barium sulfate should be avoided in certain diseases, such as gastrointestinal perforation (20). If the integrity of the gastrointestinal tract is poor, it can lead to barium peritonitis. Its clinical symptoms include abdominal palpation pain, vomiting, anorexia, drowsiness, hypotension, shortness of breath, and abdominal distension. And it can lead to some special complications, such as hypovolemia, hypoalbuminemia, severe peritonitis, multiple abdominal adhesions and granulomas, increasing the mortality rate of secondary diffuse hemorrhagic peritonitis. Barium peritonitis has a poor prognosis and a higher mortality rate than systemic sepsis peritonitis. In experimental studies, once barium sulfate is mixed with gastrointestinal contents in the abdominal cavity, the mortality rate within 24 h is 100% (21). In this case, during the clinical examination of the sick dog, no gastrointestinal symptoms such as abdominal pain or vomiting were observed, and no fluid accumulation was found on the abdominal ultrasound examination. Therefore, the possibility of gastrointestinal perforation can be ruled out. Therefore, we consider using contrast radiography (barium gavage) for clinical examination.

Treatment modalities for diaphragmatic hernia mainly include conservative and surgical treatments. Some studies have shown that surgery is mostly considered for young dogs, while most dogs considered for conservative treatment are usually not fit for surgery due to lack of obvious clinical manifestations or suffering from other diseases (22). The main accesses for diaphragmatic rupture repair include laparotomy, thoracotomy, combined thoracic-abdominal approach, and single paracostal approach. Most diaphragmatic hernias are corrected through laparotomy, which does not require consideration of the location of the diaphragmatic tear and the need for a wider field of view. The single paracostal approach is more difficult but is suitable for exposing the field of view of the dorsal diaphragmatic tear (14). The case was treated with laparotomy for diaphragmatic hernia. Therefore, the sick dog need preoperative oxygenation to improve blood oxygen concentration and ensure cardiopulmonary function. During surgery, attention should be paid to the heartbeat and blood oxygen concentration. Maintaining good mechanical ventilation and oxygenation, avoiding hypercapnia, preventing reexpansion pulmonary edema (23), and replenishing fluids and energy intravenously are recommended. The liver should be handled carefully because it is brittle and easily damaged. Before closing the hernia hole, the gas in the thoracic cavity should be aspirated with an extension tube connected to a syringe to restore the negative pressure in the thoracic cavity.

In conclusion, we reported a case of a dog with a delayed traumatic diaphragmatic hernia. According to the timeline (Supplementary Figure S2), no significant abnormality had been detected through imaging after a vehicular trauma, but the dog developed frequent coughing 6 months later. The hernia presenting with hepatothorax and enterothorax was detected through ultrasonography, X-ray, and contrast radiography (barium gavage). The hernia was corrected by suturing the ruptured diaphragm. The dog received excellent postoperative care and eventually recovered. This study provides complete contrast radiography (barium gavage) results and provides a valuable reference for the diagnosis and treatment of delayed traumatic diaphragmatic hernia.

## Data availability statement

The original contributions presented in the study are included in the article/Supplementary material, further inquiries can be directed to the corresponding authors.

## Ethics statement

Ethical approval was not required for the studies involving animals in accordance with the local legislation and institutional requirements because ethical approval was not required for the study because the case report is a description of a clinical case. Written informed consent was obtained from the owners for the participation of their animals in this study.

## Author contributions

BS: Writing – original draft, Writing – review & editing, Methodology, Conceptualization. YL: Writing – review & editing, Writing – original draft, Software, Methodology, Formal analysis, Data curation, Conceptualization. TT: Writing – original draft, Conceptualization. ZL: Writing – original draft, Conceptualization. TH: Writing – original draft, Methodology. YY: Writing – original draft, Data curation. SF: Writing – original draft, Software. SW: Writing – original draft, Methodology. HW: Writing – original draft, Conceptualization. TC: Writing – review & editing, Writing – original draft, Conceptualization. GS: Methodology, Conceptualization, Writing – review & editing, Writing – original draft.
