# Supplementary material for: Case report: Diagnosis and surgical treatment of delayed traumatic diaphragmatic hernia with hepatothorax and enterothorax in a small dog
Source: Front Vet Sci. 2024 Jun 14;11:1357626. doi: 10.3389/fvets.2024.1357626 (PMC11212120; doi:10.3389/fvets.2024.1357626)
Supplement: Supplementary file 1 [file Image_1.pdf]

Supplementary Material

Case report: Diagnosis and surgical treatment of delayed traumatic diaphragmatic hernia with hepatothorax and enterothorax in a small dog

Bing Shao†, Yiding Liu†, Tiange Tai, Zhaoyang Liu, Tianyu Han, Yu Yang, Shanshan Fei, Shu Wang, Haibin Wang

*** Correspondence:** Tiezhu Chen: cctzcd@126.com; Guangliang Shi: shiguangliang@neau.edu.cn

1 Supplementary Data

Supplementary Material should be uploaded separately on submission. Please include any supplementary data, figures and/or tables.

Supplementary material is not typeset so please ensure that all information is clearly presented, the appropriate caption is included in the file and not in the manuscript, and that the style conforms to the rest of the article.

2 Supplementary Figures and Tables

2.1 Supplementary Figures

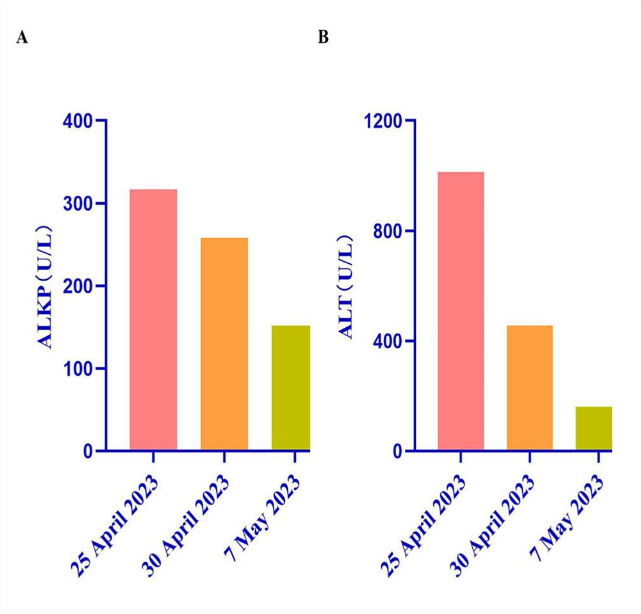

Supplementary Figure 1. Biochemical values before and after treatment.Indicators gradually converge to the normal range. **(A)**: Values of ALKP gradually decreased and normalized; **(B)**: Values of ALT gradually decreased and returned to the normal range.

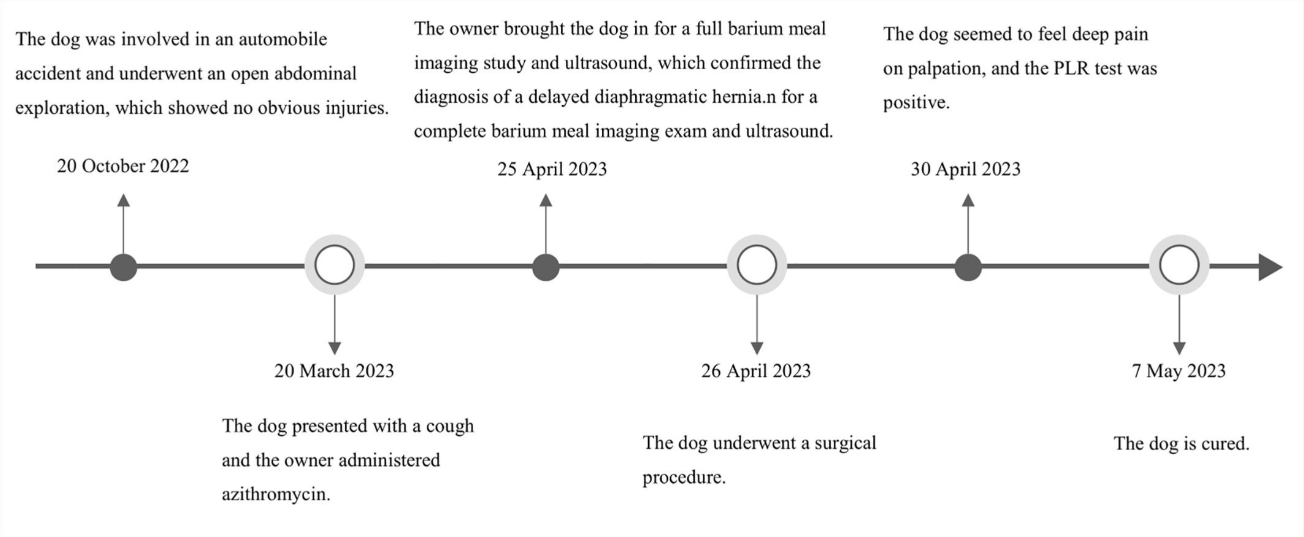

Supplementary Figure 2. Timeline of dog visits. The timeline includes the entire course of the affected dog from the time of the crash to the healing of the delayed diaphragmatic hernia.
